# Incidence, Risk-Factors and Associated Mortality of Complications in Orthogeriatric Co-Managed Inpatients

**DOI:** 10.1177/2151459321998314

**Published:** 2021-03-11

**Authors:** Andreas Wiedl, Stefan Förch, Annabel Fenwick, Edgar Mayr

**Affiliations:** 1 39694 Universitätsklinikum Augsburg, Abteilung für Unfallchirurgie, Orthopädie, Plastische und Handchirurgie, Augsburg, Germany

**Keywords:** orthogeriatric comanagement, fragility fractures, complication, mortality, risk factors, immobility, acute kidney injury, pneumonia, delirium

## Abstract

**Introduction:**

Pneumonia, thromboembolic and ischemic events, urinary tract infections (UTI), delirium and acute kidney injury (AKI) are common complications during the treatment of fragility fractures. In a 2 years-follow-up we determined the according incidence and risk factors of these and other complications in orthogeriatric inward patients, as well as the respective associated mortality.

**Methods:**

All patients treated on an orthogeriatric co-managed ward over the course of a year were included. Besides injury, therapy and geriatric assessment parameters, we evaluated the inward incidence of common complications. In a 2 years-follow-up the associated death rates were aquired. SPSS (IBM) was used to determine the importance of risk factors predisposing to the respective occurrence of a complication and accordingly determine it’s impact on the patients’ 1- and 2-years-mortality.

**Results:**

830 orthogeriatric patients were initially assessed with a remaining follow-up cohort of 661 (79.6%). We observed very few cases of thrombosis (0.6%), pulmonary embolism (0.5%), apoplex (0.5%) and myocardial infarction (0.8%). Pneumonia was seen in 42 (5.1%), UTI in 85 (10.2%), delirium in 186 (22.4%) and AKI in 91 (11.0%) patients. Consistently ADL on admission was found to be a relevant risk factor in the development of each complication. After adjustment only AKI showed a significant increased mortality risk of 1.60 (95%CI:1.086-2.350).

**Discussion:**

In our fracture-independent assessment of complications in the orthogeriatric treatment of inward patients we’ve seen very rare cases of cardiac and thrombotic complications. Typical fragility-fracture associated common events like pneumonia, UTI, delirium and AKI were still more incidental. No complication except AKI was associated to significant increased mortality risk.

**Conclusions:**

The relevance of orthogeriatric care in prevention and outcome of inward complications seems promising, needing still more controlled studies, evaluating not just hip fracture patients but more diverse groups. Consensus is needed in the scholar evaluation of orthogeriatric complications.

## Introduction

Typical fragility fractures show increasing numbers as the general age of the worldwide population and especially the industrial nations’ rises.^1-3^ Common osteoporotic fractures as hip fractures, vertebral compression fractures and humeral fractures are associated to correlated morbidity and the incidence of complications.^4^ Those are often multifactorally caused by immobility, use of indwelling bladder catheters, surgical intervention and hypovolemia.^5,6^ Typical complications in the context of orthogeriatric treatment are pneumonia, urogenital infections, kidney failure, delirium, stroke, deep venous thrombosis and pulmonary embolism.^7,8^ There are several studies investigating the development of complications associated to hip fractures, but very few highlighting other fragility fracture types. Complication being a risk factor itself, its occurrence implicates a poorer outcome in terms of survival rate. Pneumonia was identified as a significant risk factor for increased mortality after hip fractures,^9,10^ whereas urogenital infections were not observed to influence survival in a negative way.^7,11^ The occurrence of acute kidney failure and the stage of preexisting chronic kidney failure also seem to correlate with higher mortality.^12^ Delirium and associated negative consequences such as falls and noncompliance are typical in the inpatient context too.^13^ In order to address comorbidities and complications, orthogeriatric treatment was widely established. Orthogeriatric care of injured old patients has been shown to have a positive influence on treatment’s outcome.^14^

The first aim of this investigation was to determine the incidence and associated risk factors of complications of inpatients during treatment on an orthogeriatric co-managed ward. Secondly in a 2-years follow-up, respective death rates were assessed in order to calculate the association between complications and mortality.

## Methods

All inward patients treated on an orthogeriatric ward in the course of a year from February 2014 to January 2015 were assessed. Admission criteria were age >75 with typical comorbidities like impaired mobility and the necessity to use aids, dementia, acoustic and visual impairments, polypharmacy, frequent falling or sarcopenia. All patients suffered from different types of fragility fractures. There was a positive approval of the concerning institutional review board of the Bavarian state chamber of medicine on the performance of this study (Sign:7/11192). Informed consent of patients and relatives was achieved.

### Data Assessment and Follow Up

Primary assessment variables were fracture type, reason for admission, therapy and the below mentioned complications in the course of the treatment. Follow up included a timeframe of 2 years from inward treatment. Patients were addressed through questionnaires by mail. In the case of no response, patients or relatives were called with a maximum of 5 attempts. If the patient had already deceased, the exact month of death was requested from the relatives by call. Consequently, not only 1- and 2-year-mortality, but also its course could be portrayed.

### Data Analysis

SPSS (IBM) was used in our statistical analyses. We used T-tests for independent samples and the Chi square test (QST) to evaluate significant influence of the cofactors (stated below). Correlations and Odds ratios of potential risk factors were calculated. We used the Fisher’s exact test (FET) for discrimination of significant differences in mortality. The cox-regression was used to adjust and eliminate the confounding effect of the tested risk factors on mortality. Consequently, the adjusted Hazard ratio’s (HR) was calculated for each complication. In order to calculate the relative mortality risk of the cohort, we used the German statistical office’s mortality tables. The referred risks are stated as standardized mortality ratio (SMR).

### Examined Complications

Patients that suffered from pneumonia, apoplexy, deep venous thrombosis, pulmonary embolism and urogenital infections were assessed when there was clinical and diagnostical evidence (X-rays, computed tomography, sonography, urinary samples) about the event in question. Delirium was assessed by clinical aspects as well as the Confusion Assessment method. All types of delirium from active, inactive to fluctuating were summarized.

We assessed acute kidney injury (AKI) by detection of increased creatinine levels and/ or anuria, oliguria and/or related lower limb edema. We did not differentiate between the 3 stages of AKI defined by the KDIGO.

The complications were checked in the initial total patient cohort.

### Treatment

Every patient received orthogeriatric comanagement by both an orthopedic and a geriatric specialist. Patients attended daily physio- (twice) and ergotherapy. Fractures of the lower extremities generally underwent surgery, whereas fractures of the upper extremity were mostly addressed operatively (relation surgery/conservative: 5/1). Fractures of the spine were treated operatively to conservatively in a fifty-fifty ratio, the therapeutic decision depending on fracture morphology, clinical course, pain and course of radiographic changes. Rib fractures were treated conservatively. All patients were supported in finding support for their care after discharge. Those suffering from surgically treated spine fractures, lower extremity fractures and pelvic fractures received rehabilitation preferably in an inward setting. Upper extremity fracture patients were mostly advised for outward physiotherapy treatment.

### Cofactors

We evaluated age, gender, type of fracture, Parker Mobility Score (PMS), Activities of daily living (ADL), Charlson-Comorbidity-Index (CCI), preexisting dementia and sarcopenia as influential factors on the occurrence and associated mortality of the investigated complications. The fractures were summed up into 2 groups: mostly immobilizing fractures of the lower extremity, pelvis and thoracolumbar spine and mostly non immobilizing fractures of the upper extremities, ribcage and cervical spine.

## Results

Table 1 shows the general characteristics of our patients of which we assessed 830 over the course of a year with an average age of 84.5. Gender distribution displays a 3:1 ratio of women to men. The reason for admission is also listed in Table 1.

Due to loss-to follow up, we acquired follow-up data from 661 patients, resulting in a response rate of 79.6%. Table 1 also displays the general characteristics of our follow-up cohort, showing a mean age of 84.6.

**Table 1.** General Characteristics of the Total and Follow-Up Cohort.

		Total patient cohort	Follow-up cohort
Age		84.5	84.6
Gender	male	198	165
	female	632	496
Injuries	Upper extremity	119	97
	Lower extremity	387	306
	Spine	105	85
	pelvis	45	34
	Ribs	24	19
	Infections	19	15
	Multiple injuries	60	44
	Other injuries/causes of admission	71	61
	Immobilizing injury	614	483
	Non immobilizing injury	216	178

### Complication Rates

**Figure 1.**
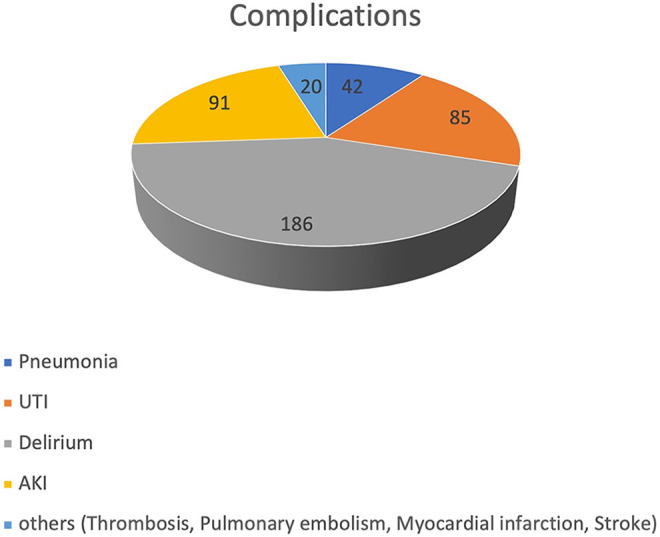
Distribution of observed complications 81x47mm (600 x 600 DPI).

**Figure 2.**
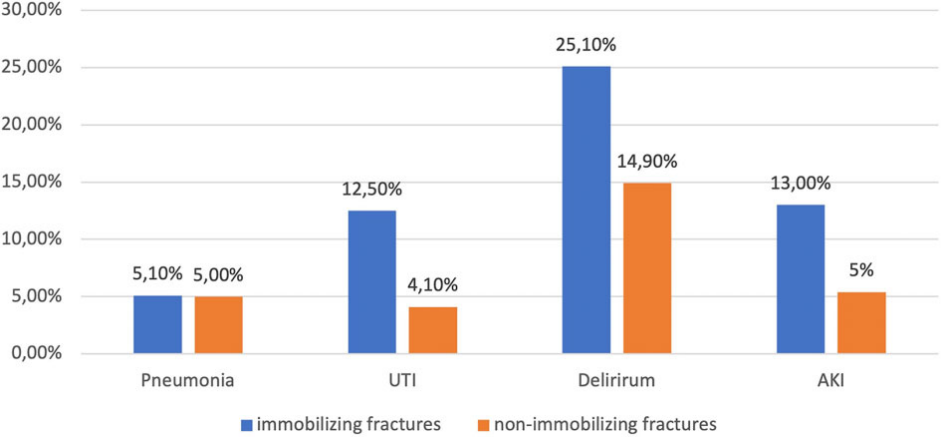
Distribution of complications among fracture types 152x88mm (600 x 600 DPI).

Overall, we found 5 (0.6%) events of thrombosis, 4 (0.5%) of pulmonary embolism (all of which occurring separately from thrombosis), 4 (0.5%) of stroke and 7 (0.8%) of acute coronary syndrome. We saw pneumonia in 42 (5.1%), urogenital infections in 85 (10.2%) and delirium in 186 (22.4%) cases. 91 (11.0%) patients developed an acute or acute on chronic kidney failure. Table 2 shows the total occurrence of complications and respective rates of the remaining patients in the follow up group. Figure 1 depicts the relative distribution of the occurred complications. Figure 2 shows their relative proportions in both fracture groups depending on immobilization. Table 3 illustrates gender, age and functional score distributions and Table 4 displays mortality values and statistical testing of the respective groups. Table 5 lists Odds-ratios for the development of the more prevalent complications in the presence of any risk factor.

**Table 2.** Total Numbers of Observed Complications.

	Total patient cohort	Follow-up cohort
Pneumonia	42	34 (81.0%)
UTI	85	60 (70.6%)
Delirium	186	142 (76.3%
AKI	91	76 (83.5%)
Thrombosis	5	3 (60.0%)
Pulmonary embolism	4	3 (75.0%)
Myocardial infarction	7	4 (57.1%)
Apoplexy	4	3 (75.0%)

**Table 3.** Age, Gender and Functional Score Distribution for each Complication.

		Age	Male	female	ADL	PMS
Pneumonia	no pneumonia	84.55(84.09-85-01)	181	607	26.55(25.33-27.76)	5.25(5.06-5.44)
	pneumonia	84.24(82-12-86.35)	17	25	18.86(15.30-22.45)	4.26(3.30-5.22)
UTI	no UTI	84.44(83.97-84.91)	186	559	26.72(25.47-27.96)	5.22(5.02-5.42)
	UTI	85.33(83.87-86.79)	12	73	21.14(17.78-24.49)	5.03(4.36-5.69)
Delirium	no delirium	83.98(83.45-84.50)	144	500	28.68(27.32-30.04)	5.43(5.22-5.65)
	delirium	86.46(85.70-87.23)	54	132	17.71(15.88-19.54)	4.40(4.03-4.76)
AKI	No AKI	84.40(83.92-84.88)	180	559	26.74(25.47-28.01)	5.24(5.04-5.44)
	AKI	85.59(84.30-86.89)	18	73	21.53(18.73-24.34)	4.90(4.32-5.47)

**Table 4.** 1- and 2-year-Mortality for Each Complication Compared to Their Reference Group.

				p-values	
		1-year-mortality	2-year-mortality	1 year	2 years
Pneumonia	no pneumonia	26.6%(167/627)	38.1%(239/627)	0.435	0.476
	pneumonia	32.4% (11/34)	44.1%(15/34)		
UTI	no UTI	26.3%(158/601)	37.1%(223/601)	0.285	0.036
	UTI	33.3% (20/60)	51.7%(31/60)		
Delirium	no delirium	24.3%(126/519)	34.3%(178/519)	0.004	0.000
	delirium	36.56%(52/142)	53.5%(76/142)		
AKI	No AKI	25.0%(146/585)	36.4%(213/585)	0.006	0.002
	AKI	42.1% (32/76)	53.9%(41/76)		

**Table 5.** Odds Ratios (OR) and p-Values Concerning the Displayed Risk Factors in the Development of the Respective Complication.

	Age	Gender	Immobilizing: Non Immobilizing fracture	ADL	PMS	Comorbidities	Dementia	Sarcopenia
Pneumonia	p = 0.767	male:female OR = 2.28(1.32-3.95) p = 0.015	OR:0.991(0.53-1.87) p = 1.000	p = 0.005	p = 0.027	OR = 1.11(0.59-2.09) p = 0.747	OR = 1.28(0.64-2.56) p = 0.588	OR = 1.01(0.54-1.89) p = 1.000)
UTI	p = 0.24	Female:male OR = 2.02(1.08-3.81) p = 0.031	OR = 3.25(1.73-6.12) p = 0.000	p = 0.005	p = 0.562	OR = 1.22(0.77-1.92) p = 0.411)	OR = 0.83(5.0-1.37) p = 0.531)	OR = 1.09(0.74-1.62) p = 0.799)
Delirium	p = 0.000	Male:female OR = 1.42 (0.98-2.05) p = 0.064	OR = 1.93(1.27-2.92) p = 0.002	p = 0.000	p = 0.000	OR = 1.14(0.81-1.59) p = 0.491	OR = 3.63(2.52-5.29) p = 0.000	OR = 1.46(1.02-2.09) p = 0.042
AKI	p = 0.104	Female:male OR = 1.31 (0.76-2.25) p = 0.364	OR = 2.79(1.58-4.92) p = 0.001	p = 0.001	p = 0.272	OR = 3.10(1.98-4.87) p = 0.000	OR = 0.79(0.49-1.29) p = 0.393	OR = 1.27(0.79-2.04) p = 0.328

Table 5  As age, PMS and ADL are continuous variables their differences in the respective complication group (complication occurring : complication not occurring) was evaluated with t-tests. For dichotomous risk factors odds ratios were calculated. This risk relation is stated respectively for gender and fracture type. Other relations: Comorbidities CCI≥4 : CCI ≤3, dementia: no dementia, sarcopnia : no sarcopenia.

### Relative Mortality Risk

We included the complications pneumonia, kidney failure, urogenital infections, delirium to the cox regression analysis. Thrombosis, stroke, acute coronary syndrome and pulmonary embolism were excluded due to small incidences that would add a bias to the analysis. After adjustment only AKI was associated to an increased death risk of 1.60. The adjusted HR’s and SMR’s are displayed in Table 6.

**Table 6.** Adjusted Hazard Ratio and Standardized Mortality Ratio Following Every Complication.

	HR	SMR
	(95%CI in brackets)	(95%CI in brackets)
Pneumonia	1.16 (0.627-2.161)	2.56 (1.266-3.854)
UTI	1.14 (0.712-1.812)	2.76 (1.787-3.733)
Delirium	1.36 (0.979-1.894)	2.53 (1.962-3.098)
AKI	1.60 (1.086-2.350)	2.87(1.991-3.749)

### Pneumonia

We saw pneumonia in 42 (5.1%) cases. Age was not found to be a significant predictor of its development. Respectively more men suffered from pneumonia (Table 3). No significant influence was seen depending on fracture related immobility (p = 1.000), whereas ADL and PMS showed significant impact on the risk of developing pneumonia (Table 4). 1-and 2-year mortality was 32.4% (11/34) a.e. 44.1% (15/34). In contrast patients not suffering from pneumonia showed corresponding values of 26.6% (167/627) and 38.1% (239/627). There was no significant difference in mortality between both groups (Table 5). The adjusted HR was 1.16 (0.627-2.161 p = 0.630). The SMR was 2.56 (95%CI: 1.266-3.854)

### Urinary Tract Infections

Urogenital infections were detected in 85 (10.2%) patients. Age did not show a relevant impact on their occurrence, whereas significantly more women suffered from UTI. Immobilizing fractures were also significantly correlated to the development of UTI (p = 0.000), as well as decreased ADL (Table 5). The associated 1- and 2-year mortality was 33.3% (20/60) and 51.7% (31/60). After 2 years the mortality was significantly higher for patients that developed a UTI. The adjusted HR was not significant being 1.14 (0.712-1.812 p = 0.592). The SMR showed a value of 2.76 (95%CI: 1.787-3.733)

### Delirium

186 (22.4%) of our patients developed any form of delirium during the course of the treatment. They were significantly older than other patients (Table 3 & 5). Gender distribution was not significantly linked to the occurrence of delirium, but a preference of men was observed. Immobilizing fractures also showed a significant higher predisposition for delirium (p = 0.002). Lower ADL, mobility and the preexistence of dementia or sarcopenia were also significantly correlated to its occurrence (Table 5). 1- and 2-year-mortalitiy was significantly raised for patients suffering from delirium, showing values of 36.6% (52/142) and 53.5% (76/142), but after adjustment, the HR just showed an increased but not significant value of 1.36 (0.979-1.894 p = 0.066). The SMR was 2.53 (95%CI: 1.962-3.098)

### Kidney Failure

91 (11.0%) patients developed acute kidney injury. No significant influence of age or gender was found in the distribution of patients with AKI. Immobilizing injuries significantly correlated with the occurrence of AKI (p = 0.000), as well as low ADL on admission and preexisting higher CCI. Both 1- and 2-year mortality of patients who developed AKI was significantly higher in contrast to those who did not, showing values of 42.1% and 53.9%. The adjusted HR was significant being 1.60 (1.086-2.350 p = 0.017). The SMR for AKI was 2.87(95%CI: 1.991-3.749).

## Discussion

The goal of this study was to determine incidences and risk factors of common complications associated to inpatient orthogeriatric treatment of fragility fractures and their impact on the patients’ mortality. Data was assessed over the course of a year including 830 patients on a co-managed orthogeriatric ward. By including different fracture types, this investigation is different to the majority of existing studies concerning the topic, which were performed on hip fracture patients exclusively.^8^ Another difficulty in the comparison of studies evaluating complications in the orthogeriatric context is the missing consensus of the relevant highlighted complications.^15^

We observed very few numbers of thrombosis, pulmonary embolism, stroke and myocardial infarction, which did not predispose for a reasonable statistical analysis. Knobe et al. confirm our results by having seen a remarkable reduction in cardiorespiratory events in co-managed orthogeriatric inpatients compared to conventional traumatological treatment in a pilot study.^16^ We’ve seen delirium in every fifth, UTI and AKI in every tenth and pneumonia in every twentieth patient. In the univariate analysis UTI, delirium and AKI were associated to higher death-rates. After correction for confounding risk factors, AKI was the only complication followed by a significant higher mortality risk. All SMR’s showed more than twicely increased death risks by any complication compared to the age-adjusted healthy population.

We’ve found male gender, ADL and mobility as independent risk factors in the development of pneumonia. Its occurrence was independent from the immobilizing character of the fracture type. Roche et al. and Lv et al. describe male gender, age, number of comorbidities and preexisting respiratory disease as according risk factors.^9,10^ No significantly higher mortality was seen in patients suffering from pneumonia. This is contrary to the existing literature.^9,10^ A possible explanation is that the mentioned studies analyzed cohorts from 2000-2011^10^ and 1999-2003.^9^ Being not specific about the treatment, these periods are prior to the implementation of orthogeriatric co-management, which could have been beneficial for patients in our investigation.

Besides mostly women suffering from UTI, main risk factors for its occurence were immobilizing fracture and reduced ADL. This seems plausible, using indwelling catheters more frequently in women and more prolonged in the context of immobilization and reduced capability of self-care. In a meta-analysis catheter use, its duration and female gender were main risk factors among others in the development of UTI.^17^ We found UTI to be associated with significantly higher mortality after 2 years of observation. This has not been reported in literature before. Hedström et al. and Hälleberg et al. detected no increased death rates after the occurrence of UTI in their hip fracture cohorts.^7,18^ We have yet seen a significant difference in survival in the late observation period, detecting none in the first year of follow up, which could have been caused by concurring confounding circumstances not necessarily associated to UTI. This assumption is supported by the adjusted HR not being significantly raised.

Delirium was prevalent frequently in our cohort, showing correlations with age, fracture type, impaired ADL, mobility, cognition and preexisting sarcopenia. Age, dementia and decreased functional status were consistently reported to be relevant risk factors in the development of delirium^19^ after hip fractures, also including acoustic and visual impairments, infections, comorbidities e.g. as renal failure, diabetes and Parkinson’s disease.^13,20^ Kim et al. described preoperative urinary catheter insertion and polymedication as independently associated to its occurrence.^5^ We detected a significant correlation of higher mortality rates in our patients suffering from delirium. This association is widely confirmed by Nightingale et al., de Jong et al. and Mitchell et al.^19,21,22^ Delirium being generally correlated to poorer health status, higher age, less cognition and predisposition to consequent injuries, it seems a legitimate indicator of higher susceptibility to earlier decease. Although after adjustment the HR was not significant, it was distinctly raised.

AKI was correlated with immobilizing injuries as well as lower ADL and more preexisting comorbidities measured by CCI. In association to the first mentioned, we must suppose that these fractures were more often treated surgically, and these surgeries were more often correlated with higher loss in volume than those addressing non-immobilizing fractures. Ulucay et al. detected lower glomerular filtration rate as an according independent risk factor, whereas Bennet et al. found male sex, vascular diseases, hypertension, diabetes, chronic kidney disease and use of nephrotoxic medication to be independently correlated to AKI following hip fractures.^23,24^ This applies to our analysis as comorbidity-associated risk factors mentioned by Bennet et al. like preexisting chronic kidney disease and diabetes are covered by the CCI. In patients suffering from AKI we’ve seen significantly increased mortality even after correction for confounders. We did not differentiate between the 3 KDIGO stages of AKI and therefore a stage-dependent risk stratification could not be delivered. Pedersen et al. examined a cohort of over 10000 patients suffering from hip fractures for the incidence of AKI, which was found to be 12.7%. The associated death rate was increased showing a 1-year-mortality of 25% and it correlated with the according KDIGO stage.^25^

The orthogeriatric approach is promising regarding the management and prophylaxis of fragility fracture-associated complications. However, studies have shown sobering results, Kusen et al. and Abrahamsen et al. found no significant reduction in complication rates comparing orthogeriatric to conventional care.^14,26^ Knobe et al. showed distinct insignificant reductions in cardio-respiratory complications in their orthogeriatric cohort, which is similar to our observation of very low rates of pulmonary embolism, myocardial infarction and stroke.^16^

We could examine an entire orthogeriatric cohort over the course of a year, deliver complication rates and determine relevant risk factors, as well as the associated 1- and 2-year-mortality. We examined the entirety of fragility fracture entities treated on an orthogeriatric ward, which is unique compared to the existing studies mainly performed on patients suffering from hip fractures.

## Limitations

This study was not performed in comparison to a conventionally treated control group, we could therefore not measure the effect of orthogeriatric treatment on the complication rate. Our cohort might be too inhomogeneous for a detailed comparison to other study samples, especially having included different types of injuries. We tried to assess the main relevant complications in the context of fragility fractures, due to the missing consensus as mentioned above, there is a wide variety of risk factors and observed complications in literature, that we did not cover in its completeness. We also missed out in differentiation of AKI stages and different types of delirium for a more detailed stratification.

## Conclusion

Pneumonia, UTI, delirium and AKI are relevant complications occuring in the treatment of fragility fractures. We could find ADL on admission to be throughout a significant risk factor. AKI had a significant, delirium a distinct but not significant impact on mortality after correction for confounders in our analyses. We observed very low rates of thrombosis, pulmonary embolism, myocardial infarction and stroke. The importance of orthogeriatric care in the prevention and treatment of complications has still to be evaluated, showing reduction of incidences but not yet delivering significant results. Finally, consensus must be found in determining which specific complications should be systematically evaluated in the scholar assessment of orthogeriatric outcome.
